# Enhancing customer retention in telecom industry with machine learning driven churn prediction

**DOI:** 10.1038/s41598-024-63750-0

**Published:** 2024-06-07

**Authors:** Alisha Sikri, Roshan Jameel, Sheikh Mohammad Idrees, Harleen Kaur

**Affiliations:** 1 Noida Institute of Engineering and Technology, Greater Noida, 201306 Uttar Pradesh India; 2 Westford University College, Sharjah, United Arab Emirates; 3 Department of Computer Science (IDI), Norwegian University of Science and Technology, Trondheim, Norway; 4 Department of Computer Science, Jamia Hamdard, New Delhi, India

**Keywords:** Customer churn, Machine learning, Data Balancing, Prediction, Ensemble, Data resampling, Energy science and technology, Aerospace engineering, Electrical and electronic engineering

## Abstract

Customer churn remains a critical concern for businesses, highlighting the significance of retaining existing customers over acquiring new ones. Effective prediction of potential churners aids in devising robust retention policies and efficient customer management strategies. This study dives into the realm of machine learning algorithms for predictive analysis in churn prediction, addressing the inherent challenge posed by diverse and imbalanced customer churn data distributions. This paper introduces a novel approach—the Ratio-based data balancing technique, which addresses data skewness as a pre-processing step, ensuring improved accuracy in predictive modelling. This study fills gaps in existing literature by highlighting the effectiveness of ensemble algorithms and the critical role of data balancing techniques in optimizing churn prediction models. While our research contributes a novel approach, there remain avenues for further exploration. This work evaluates several machine learning algorithms—Perceptron, Multi-Layer Perceptron, Naive Bayes, Logistic Regression, K-Nearest Neighbour, Decision Tree, alongside Ensemble techniques such as Gradient Boosting and Extreme Gradient Boosting (XGBoost)—on balanced datasets achieved through our proposed Ratio-based data balancing technique and the commonly used Data Resampling. Results reveal that our proposed Ratio-based data balancing technique notably outperforms traditional Over-Sampling and Under-Sampling methods in churn prediction accuracy. Additionally, using combined algorithms like Gradient Boosting and XGBoost showed better results than using single methods. Our study looked at different aspects like Accuracy, Precision, Recall, and F-Score, finding that these combined methods are better for predicting customer churn. Specifically, when we used a 75:25 ratio with the XGBoost method, we got the most promising results for our analysis which are presented in this work.

## Introduction

Telecommunication is a highly competitive industry. There a several runners in the market, thus managing relationship with the customers has become vital for the service providers^1^. The organizations employ a variety of tactics to increase their revenues such as attracting new customers, selling more services to the existing customers and most importantly retaining the old customers^2^. Customer churn is a situation in which an existing customer leaves the services of a particular provider. It can be conceptually categorized in two ways. The first one is desire of customer to switch to a new service provider^11^ and second one is the appeal of customer to stop using the services of the current provider^12^. If a large number of customers churn in a short span of time, the reputation of the provider gets affected^7^, as the businesses nowadays are highly affected by the word of mouth and social media influence^8^. The issues related to the customer support and service satisfaction are the main reasons behind the churn. Working on preventing the existing customers from churning is inexpensive in terms of cost and time both and keeps the performance of the firm stable and strong^3,4^. Captivating the new customers is said to be around five times costlier that stopping the existing ones to leave^5,6^. Consequently, a shift in the marketing strategies is being noticed as the organizations are focusing on retaining the existing customers then acquiring the new ones. There are two ways this retention of the existing customers could be done. The first way is to provide improved customer service and loyalty, running campaigns, bestow good offers etc.; thus, enhancing their experience and making them stay for longer period of time. However, this approach is not very feasible and cost effective as serving a huge number of customers with such facilities is not an easy task. Therefore, the second approach could be applied i.e., predicting the possible churners and focusing on them with effective retention strategies^9^.

The goal of the churn prediction approaches is to identify the early signs of the possible churners by analyzing the existing information the providers have, about their customer’s behavior^10^. The customer churn can be protected in two ways^2^. The first one could be the “reactive approach” in which the provider’s does not analyze anything beforehand, they only come into action when the customer has already filed for the cancellation of the subscription or requests for porting to a new provider. Then the provider tries to lure the customer by providing them exciting offers and discounts. However, this approach rarely works. The second way to handle the situation is the “proactive approach” that is done to predict the possibility of the customers to churn. It is very crucial to analyze the behavior of the customers to timely and accurately predict the possible churners. With the growth in the field of machine learning and data analytics, the prediction of customer churn is becoming an in-demand topic of analysis in the field of both computer science as well as marketing. The researchers are tackling the customer churn problem with the help of several machine learning techniques, as they have the capability of analyzing and predicting the upcoming events on the basis of existing information^13,14^. There are several machine learning approaches such as Support Vector Machine (SVM), Logistic Regression, Naïve Bayes, Artificial Neural Network (ANN) etc. and ensemble approaches such as boosting algorithms etc. that have proved their effectiveness in classification and prediction related problems. Although, a very few of these algorithms have been applied to predict the customer churn.

There are numerous elements that makes the prediction of customer churn using machine learning techniques difficult. Imbalanced data is one such factor that is usually noticed in the customer churn datasets that affects the prediction accuracy. Imbalanced data is a problem with the dataset where there is skewed proportion of the target variables i.e., the size of the classes have huge difference. In such situations one of the classes is having a large number of samples called the “Majority Class” whereas, the other one has lesser number of instances called “Minority Class”. This leads to the incorrect learning of the algorithms and hence, giving the incorrect results. The imbalanced data is a problem that has been reported in many application domains such as fraudulent transaction detection, medical diagnosis, text classification etc. It can cause a huge problem in case of customer churn prediction, because if the algorithm wrongly predicts a loyal customer as a churner, then it will be a waste of efforts, whereas, if a churner is wrongly misidentified as a loyal customer, then it will be a loss of a customer. Therefore, the data should be pre-processed and balanced before applying the machine learning techniques to make sure that the results achieved are unbiased and accurate.

In this work, we are trying to predict the customer churn on the dataset downloaded from Kaggle^15^. In order to handle the imbalanced nature of this dataset, we have proposed a novel approach called Ratio based data balancing. Then, several standalone machine learning algorithms such as Perceptron, Multi-Layer Perceptron, Naive Bayes, Logistic Regression, K-Nearest Neighbor, Decision Tree, and Ensemble techniques namely Gradient Boosting and Extreme Gradient Boosting (XGBoost) are applied to predict the customer churn. We have proved the effectiveness of our proposed technique by comparing the predictions made by the abovementioned machine learning algorithms on the dataset balanced with our proposed Ratio based data balancing technique and balanced by the most widely used data balancing technique called Data Resampling.

The contributions of this research work can be summarized as follows:

- To understand the impact of Imbalanced dataset on the performance of Machine learning algorithms.
- To propose a novel Ratio based data balancing technique for handling the customer churn dataset.
- Apply several standalone and ensemble machine learning techniques to predict the customer churn on imbalanced dataset.
- Apply several standalone and ensemble machine learning techniques to predict the customer churn on balanced dataset.
- Compare the effectiveness of the proposed Ratio based data balancing with the most widely adopted data balancing technique called Resampling in terms of various performance metrics.

The rest of this article is organized as follows, the next section, Section "Related work" discusses about the existing works in the field of customer churn prediction problem, followed by the methodology opted for carrying out this work discussed in Section "Methodology", the interpretation of results is done in Section "Result interpretation", the conclusion of the findings is done in Section "Conclusion and future scope".

## Related work

For any business the customers can be of two types: the first ones are those who are using the services in a pre-paid manner i.e., they are having some subscription of the services and are availing those subscriptions. While the second ones are the post-paid customers who are using the services and paying for the used services later^7^. It is easy to predict the churn from the first category as the services are taken beforehand and if a customer cancels the subscription, then a potential churn can be identified. However, in the latter category, the customer can stop using the services without any previous signal or information. In this research work, we are focused on the customers belonging to the first category i.e., subscription-based category in tele-communication industry. As the telecommunication industry is highly competitive in nature and there are various businesses in the market trying to lure the customers with exciting offers and benefits. Thus, it becomes necessary for the companies to hold on to the existing customers by correctly predicting the possible churners. This could be achieved using data analytics and machine learning techniques^16^. The customer churn prediction comes under the classification problem in which the service providers are supposed to classify the churners and no-churners among the customers based on the existing information about their service usage. The classification comes under the supervised machine learning category. Therefore, supervised machine learning techniques can be leveraged to identify and predict the potential churners^17^.

The effectiveness of the machine learning techniques for prediction of the customer churn has been analyzed in several studies^14^. Different authors have applied various machine learning techniques to predict the possible customer churn, however, no single technique has yet been identified to be the best one for the problem^18^. The researchers have surveyed various articles based on machine learning to predict the customer churn in order to determine the best approach to conduct the analysis. A survey of 61 research papers was done in^19^ the authors reviewed the publications made during 2002–2013 in journals and found that the most widely used algorithms during that period were found to be Logistic Regression, Neural Network and Decision Tree. Another review conducted by the authors of^20^ identified Logistic Regression, Naïve Bayes, SVM, ANN and Decision Tree to be the most prominently used algorithms during the period of 2014–2017. A comparative study performed^21^ suggested SVM, Naïve Bayes and Multi-layer neural network attained the maximum accuracy. The SVM was also suggested as the best performer by^22^. Whereas, the analysis conducted by^23,24^ advocated the accuracy of Neural Network to be higher than any other machine learning technique.

A comparative study of machine learning techniques for predicting customer purchasing behavior, including logistic regression, decision tree, k-nearest neighbors (KNN), Naïve Bayes, SVM, random forest, stochastic gradient descent (SGD), ANN, AdaBoost, XgBoost, and dummy classifier is presented in^55^. Hybrid algorithms using stacking, such as SvmAda, RfAda, and KnnSgd, are also explored. The best-performing model is identified as the hybrid classifier KnnSgd, achieving an accuracy of 92.42%, with the paper attributing its success to minimizing errors through a combination of KNN and SGD. The ensemble machine learning techniques have gained a lot of popularity in the past few years. The ensemble techniques work by combining multiple existing machine learning algorithms so as to achieve better predictive results. The ensemble techniques can be categorized into two types: Bagging and Boosting. The Random Forest algorithm comes under the bagging category whereas the Gradient Boosting, Light GBM, Extreme Gradient Boosting (XGBoost) etc. comes under the boosting category. Several researches and surveys conducted in the past suggests that the ensemble algorithms outperform the standalone techniques with a marginal difference^16,22^. The boosting and bagging algorithms have proved their effectiveness in various application domain; however, they have not very much used in the field of customer churn prediction. XGBoost is an ensemble technique which is an extension of Gradient Boosting algorithm^25^. It has now become the first choice of the researchers working with ensemble techniques and has proved its effectiveness on various applications such as diagnosis of diseases, analysis of health records, metagenomics, credit card frauds etc.^18^. It has been proved by several researchers that XGBoost is the most accurate algorithms out of all the standalone and ensemble techniques and has outperformed in various application areas including intrusion detection^26,35^, credit card fraudulent payments^27^, mobile fraudulent payment detection^28^ etc. to name a few. The XGBoost has also been applied to the customer churn prediction problem and has attained accuracy and F-score better than the bagging technique Random Forest and standalone technique KNN^29^. Another study^2^ suggested the ROC-AUC score achieved by the XGBoost was higher than traditional standalone machine learning techniques as well as other ensemble algorithms.

A novel adaptive learning approach for Customer Churn Prediction (CCP) in the telecommunications industry is proposed in^51^, that leverages the Naïve Bayes classifier with a Genetic Algorithm-based feature weighting strategy. The proposed method demonstrates superior predictive performance on publicly available datasets, including BigML Telco, IBM Telco, and Cell2Cell. Another study^52^ introduces an intelligent rule-based decision-making technique based on rough set theory. The authors claim that their proposed method effectively classifies churn and non-churn customers. Through extensive simulation experiments, the authors demonstrate that the rough set approach, particularly using the Genetic Algorithm, outperforms other rule-generation mechanisms. The study concludes by emphasizing the potential of attribute-level analysis for informing successful customer retention policies in the telecom sector, contributing to strategic decision-making processes. Article^53^ proposes a Just-In-Time (JIT) approach for Customer Churn Prediction (CCP), focusing on cross-company prediction. To bridge the gap, the authors introduce a JIT-CCP model using cross-company data and evaluate the impact of state-of-the-art data transformation methods on its performance. The experiments, conducted on benchmark datasets using Naive Bayes as the underlying classifier, reveal that data transformation methods significantly enhance the JIT-CCP model's performance, demonstrating its superiority over models without such transformations.

The customer churn data is high-dimensional in nature, authors in^54^ highlighted the issues of noise, computational complexity, and information loss during feature reduction in preprocessing phase. A novel feature weighting technique is proposed in this paper using a genetic algorithm to automatically assign weights to attributes based on Naïve Bayes classification. Experiments on a publicly available dataset in the telecommunications sector demonstrate that the proposed approach achieves superior performance, with an overall accuracy of 89.1% and precision of 95.65%, showcasing the effectiveness of the technique in predicting customer churn.

The datasets used for the prediction of customer churn are usually imbalanced in nature and data balancing techniques are required to be applied. An imbalanced data is a type of dataset in which the distribution of one of the classes is more than the other one^30^. In such situations one of the classes is having a large number of samples called the “Majority Class” whereas, the other one has lesser number of instances called “Minority Class”. This leads to the incorrect learning of the algorithms and hence, giving the incorrect results^31^. Therefore, data balancing techniques must be applied to rebalance the dataset and improve the classification accuracy of the machine learning techniques applied afterwards^32,33^. An argument is presented on the adversity of the imbalanced data in^34^, where the authors identify the reasons why a classification algorithm mis predict the outcomes because of the imbalanced dataset. They claimed that the accuracy achieved is higher even after the misclassified outputs is because of the low distribution of the minority class in the dataset. They also argued that the algorithm neglects the minority class by considering them as noise. There are various application domains that faces the problems associated with the imbalanced dataset including the telecom customer churn, in which the churners are closely related to non-churners, thus making it difficult for the predicting algorithms to correctly identify and prevent the possible churning.

The “Data Level Approaches” are most commonly used for handling the imbalanced datasets. The resampling of the dataset is done in this approach either the Minority cases data entries are added to the dataset or the Majority cases data entries are removed to even out the distribution of both the classes. The removal of Majority cases is known as “Under-sampling” whereas, adding the Minority cases is called “Under-sampling”^41,42^. Since, the under-sampling could lead to the loss of vital data entries, it is recommended to use the over-sampling for handling the imbalanced dataset. Although, by duplicating the Minority class samples in over-sampling, the learning efficiency of the algorithms is compromised. Because the training model tends to over-fit because of the additional information and data entries provided to the algorithm^43^.

## Methodology

**Figure 1:**
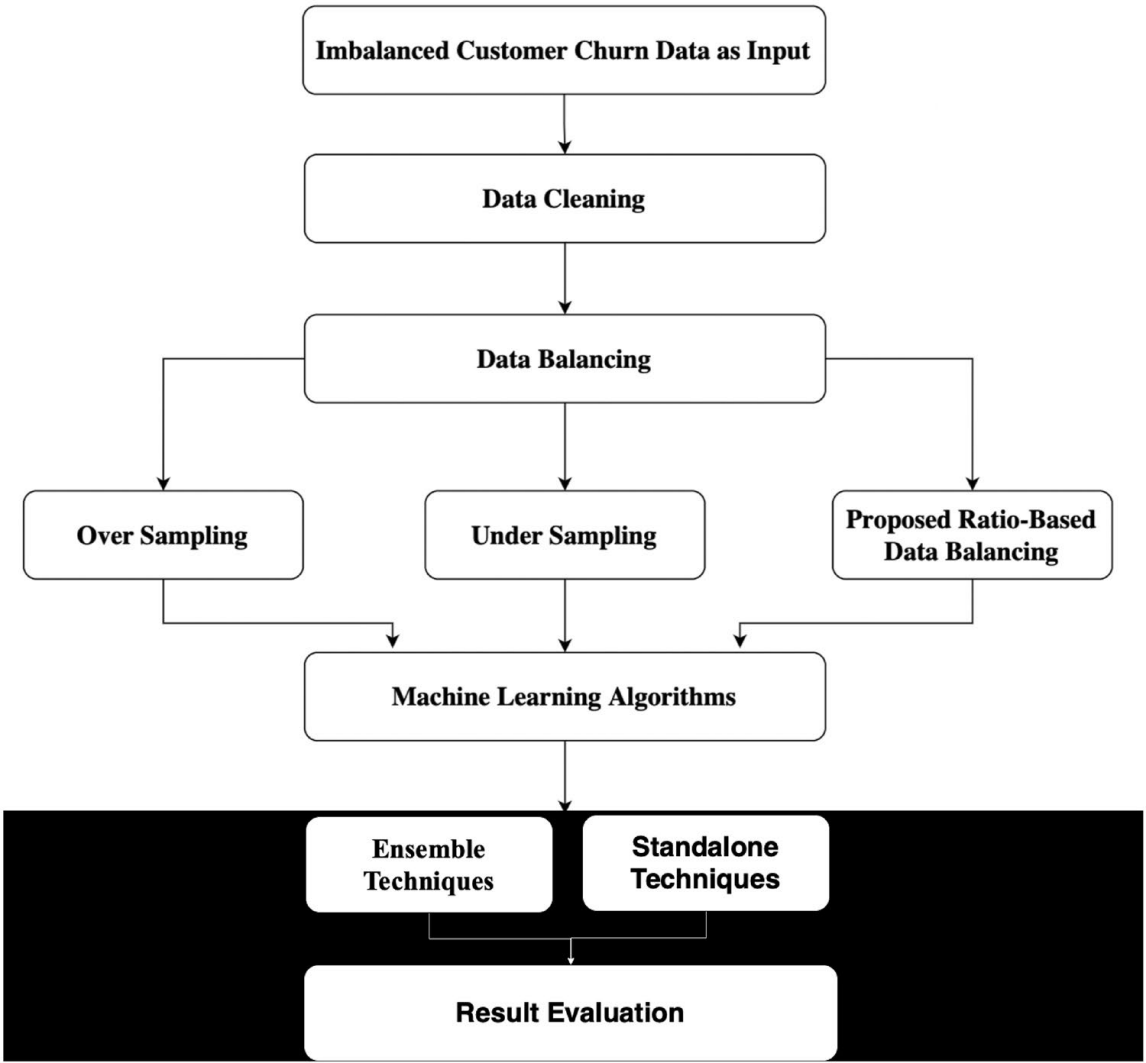
Customer churn prediction framework.

The approach of the research conducted for this paper is represented in the Fig. 1 below. The customer churn in telecommunication industry dataset is first extracted^57,58^. Then, the preprocessing of the data is performed. The data cleaning is performed first, and then the customer churn prediction is done on imbalanced data by applying various Machine learning standalone and ensemble techniques. Then, the data is balanced using existing resampling techniques, Over Sampling and Under Sampling and our proposed Ratio Based Data Balancing technique. The Machine learning techniques are then applied on the balanced dataset extracted from the three algorithms. The performance of the Machine Learning based prediction is then evaluated using multiple metrics for imbalanced as well as balanced datasets. The goal of the research is to evaluate the impact of imbalanced data on prediction of customer churn by comparing the results achieved on balanced and imbalanced data. And to compare the existing techniques available for data balancing and the proposed approach.

### Dataset description

The dataset we used for our research consists of 20 features including the target variable. The first 19 attributes consists of the information related to the customer, whereas the 20^th^ attribute is the target variable that is used to point out the person is a churner or not. A detailed description of the dataset and attributes is given in Table 1 below.

**Table 1:** Dataset description.

S. No	Name of the attribute	Description	Type
1	state_code	State code of the customer	String
2	account_length	Number of months the customer is associated with the current service provider	Numerical
3	area_code	Area code of the customer	String
4	international_plan	Whether the customer has international pan	Categorical
5	voice_mail_plan	Whether the customer has voice mail pan	Categorical
6	number_vmail_messages	Number of voice mail messages	numerical
7	total_day_minutes	Total minutes of calls made during the day time	Numerical
8	total_day_calls	Total number of calls made during the day time	Numerical
9	total_day_charge	Total charge of the calls made during the day time	Numerical
10	total_eve_minutes	Total minutes of calls made during the evening time	Numerical
11	total_eve_calls	Total number of calls made during the evening time	Numerical
12	total_eve_charge	Total charge of the calls made during the evening time	Numerical
13	total_night_minutes	Total minutes of calls made during the night time	Numerical
14	total_night_calls	Total number of calls made during the night time	Numerical
15	total_night_charge	Total charge of the calls made during the night time	Numerical
16	total_intl_minutes	Total minutes of international calls	Numerical
17	total_intl_calls	Total number of international calls	Numerical
18	total_intl_charge	Total charge of international calls	Numerical
19	number_customer_service_calls	Number of calls made to the customer service	Numerical
20	Churn	Customer churn	Categorical

### Data cleaning

As mentioned in the dataset description above, the target variable is in categorical form i.e., Yes/No, where Yes denotes the customer churn while No depicts that the customer did not churn. We have converted the categorical data to Binary by replacing Yes to 1 and No to 0. Furthermore, we realized that the attributes state_code, area_code and account_length does not have any significance in prediction of customer churn, therefore we have dropped these attributes at the time of implementing the machine learning algorithms.

### Data balancing

There are four ways to deal with the imbalanced datasets^36,37^. First is “Data Level Approach” which is the most widely used approach, in which the data is balanced by “Resampling” techniques. The Second approach is “Algorithm Level Approach” that modifies the machine learning algorithms being applied for the classification and make them consider the minority data instances. Third approach is “Cost-Sensitive Learning Approach” that handles the imbalanced data by assigning the misclassification costs^38^. The Fourth approach is “Classifier Ensemble Techniques” that constructs the ensemble classification algorithms to calculate the final outcomes. Some of the researchers have even combined the two of the approaches such as the Data Level Approach and the Classifier Ensemble Technique to effectively promote the accuracy of classification algorithms^39^. Of all the above-mentioned approaches the Data Level Approaches are the most widely accepted and used for customer churn predictions as they are easy to implement and requires less time for computation^40^. We have also used the Resampling technique to balance the dataset and compare the results achieved by our proposed technique.

#### Over sampling

In this resampling approach of balancing the dataset, the data samples are added to the minority class so that the skewedness in the distribution because of the majority data class can be decreased. In this approach a number of entries with Minority distribution are added to the dataset to make the values of the target variable equal for both the classes.

#### Under sampling

In this resampling approach of balancing the dataset, the data samples are removed from the majority class so that the percentage of the minority data values in the overall distribution of the data is made equal to the values of data belonging majority class.

#### Proposed ratio-based data balancing

The proposed technique works by adjusting the target variable in a fixed ratio, i.e. the Yes and No values in the training dataset are taken in fixed amount. We have taken five combinations of the ratio (i) 90:10 (ii) 80:20 (iii) 75:25 (iv) 65:35 and (v) 50:50 and performed the classification by applying several machine learning techniques.

### Machine learning algorithms for customer churn prediction

The dataset was divided into 75:25 for training and testing and the machine learning techniques are applied to predict the Customer churn on the balanced and imbalanced telecommunication dataset. A total of ten standalone and ensemble techniques^44–50^ are implemented to evaluate the performance of the existing and proposed data balancing techniques. A brief of the algorithms implemented in this research work is given below:

#### Perceptron

It is also a supervised machine learning technique that classifies the data on the basis of Artificial Neural Network (ANN). It is the simplest type of ANN that consists of a single layer with four parameter (i) input (ii) weight (iii) output and (iv) activation function. It is also called a binary classifier as it divides the data into two classes.

#### Multi-layer perceptron (MLP)

It is also a neural network-based machine learning technique. It is a dense network that has multiple layers connected densely and can convert any dimensional input data to the output with preferred dimensions. In MLP the nodes are connected to form a network such that output of one layer is input to the next one.

#### Naive bayes

Naïve Bayes algorithm is made up of two words (i) Naïve that means the existence of one feature is not dependent on other features and (ii) Bayes means it works on the concept of Bayes Theorem which calculates the probability of an event on the basis of existing information. This algorithm is best suited for text data classification.

#### Logistic regression

It is one of the most famous Supervised Machine learning algorithms. It can be used for both classification and regression related scenarios or in other words, it can be considered as a linear regression algorithm for classification of categorical target values^56^. It can be used to predict the dependent variable by using the independent variables. It is based on the concept of “Maximum Likelihood”, it gives the output as a categorical value either Yes/No or 0/1 on the basis of probability.

#### K-nearest neighbor (KNN)

KNN is the simplest Supervised Machine learning technique that takes the similarity of the test data input values and the values of the existing class members into consideration and classifies it on the basis of similarity. It is also known as lazy-learner as it does not learn during the training phase, rather it stores the dataset and classify the new values at the time of classification only.

#### Decision tree

The decision tree algorithm also falls under the supervised machine learning techniques. It is represented in the form of a tree in which the nodes of the tree represent the attributes of the data, edges depict the decision or possible solutions available and the end nodes called the leaf nodes represents the outcome. Basically, it is a graphical representation of all the attributes and possible outcomes of the algorithm.

#### Gradient boosting

The boosting algorithm works as a model is designed and implemented first, and then a second model is implemented to correct the inaccuracies in the first one. The basic principle is to align models in sequential manner, each model correcting the errors of the previous ones. In Gradient Boosting, the focus is on minimizing the mean squared errors of the loss.

#### Extreme gradient boosting (XGBoost)

The XGBoost is a prominent supervised machine learning technique for handling the classification, regression and rank based problems. It is an implementation of Gradient Boosting applied on the Decision Trees. In this approach the decision trees are implemented in sequential manner.

## Result interpretation

The framework followed in this research has taken the customer churn data of telecommunication industry. The goal of this work is to analyse the impact of the imbalanced dataset on the predictions made by the machine learning classification algorithms. We have first taken the imbalanced data as it existed and applied eight machine learning algorithms mentioned in the previous sections to that dataset and the results are depicted in Table 2 below. Next, the dataset is balanced using three methods namely “Under-Sampling”, “Over-Sampling” and the proposed “Ratio-based data balancing” individually. After balancing the dataset the Machine Learning techniques are applied and various performance metrics are calculated to compare the performance of the algorithms on the dataset balanced using different techniques. The results are shown below in Tables 3, 4, 5, 6, 7, 8 and 9. The parameters used in this work for the performance measurement are as follows:

- *Accuracy:* It is the most important performance metrics, it gives the total number of correct predictions made divided by the total number of classified values.
- *Precision:* It can be defined as the total number of correct predictions made by the classifier among the total number of positive predictions.
- *Recall:* It gives the value of total number of predictions made correctly by the classification algorithm divided by the total number of actual positive values.
- *F1-Score:* It is a weighted average of Precision and Recall.

**Table 2:** Performance metrics for imbalanced dataset.

	Perceptron	MLP Classifier	Naïve Bayes	Logistic Regression	KNN	Decision Tree	Gradient Boosting	Extreme Gradient Boosting
Accuracy	78.04	73.12	77.87	78.56	74.72	79.6	82.4	83.68
Precision	0	54.54	49.13	33.33	64.38	46.42	60	74.02
Recall	0	2.83	40.09	28.3	22.16	55.18	24.05	49.97
F1-Score	0	5.38	44.15	30.61	32.98	50.43	34.34	59.66

**Table 3:** Performance metrics for balanced dataset using over sampling approach.

	Perceptron	MLP Classifier	Naïve Bayes	Logistic Regression	KNN	Decision Tree	Gradient Boosting	XG-Boosting
Accuracy	44.08	77.04	76.72	70	62.48	83.92	77.2	84.48
Precision	21.09	33.83	40.28	33.33	25.05	54.66	40.86	75.32
Recall	**78.37**	30.63	64.41	68.91	55.85	55.40	63.51	52.25
F1-Score	33.23	32.15	49.56	44.93	34.58	55.03	49.73	61.70

**Table 4:** Performance metrics for balanced dataset using under sampling approach.

	Perceptron	MLP Classifier	Naïve Bayes	Logistic Regression	KNN	Decision Tree	Gradient Boosting	XG-Boosting
Accuracy	44.08	79.76	76.96	68.08	63.52	65.04	76.16	77.76
Precision	21.09	42.72	40.83	31.75	25.82	29.04	39.50	47.08
Recall	78.37	40.99	66.21	69.36	56.30	67.11	64.41	60.72
F1-Score	33.23	41.83	50.51	43.56	35.41	40.54	48.97	53.04

**Table 5:** Performance metrics for balanced dataset using ratio based data balancing approach with 90:10 ratio.

	Perceptron	MLP Classifier	Naïve Bayes	Logistic Regression	KNN	Decision Tree	Gradient Boosting	XG- Boosting
Accuracy	84.8	83.84	82.64	84.64	85.28	84.80	85.28	88.72
Precision	72.72	47.05	42.46	70.37	70.37	51.46	80.00	75.96
Recall	4.10	28.71	31.79	9.74	9.74	45.12	36.92	40.51
F1-Score	7.76	35.66	36.36	17.11	17.11	48.08	50.52	52.84

**Table 6:** Performance metrics for balanced dataset using ratio based data balancing approach with 80:20 ratio.

	Perceptron	MLP Classifier	Naïve Bayes	Logistic Regression	KNN	Decision Tree	Gradient Boosting	XG-Boosting
Accuracy	17.52	85.52	82.80	84.08	84.88	80.88	85.20	89.28
Precision	15.73	65.21	44.38	35.71	53.94	40.98	78.86	72.59
Recall	98.46	15.38	40.51	2.56	21.02	51.28	49.74	50.25
F1-Score	27.13	24.89	42.35	4.78	30.25	45.55	61.00	59.39

**Table 7:** Performance metrics for balanced dataset using ratio based data balancing approach with 75:25 ratio.

	Perceptron	MLP Classifier	Naïve Bayes	Logistic Regression	KNN	Decision Tree	Gradient Boosting	XG- Boosting
Accuracy	62.24	79.76	82.56	83.60	82.56	79.20	84.48	89.60
Precision	20.46	38.01	44.22	41.07	40.17	38.90	76.42	76.04
Recall	49.23	47.17	45.12	11.79	24.10	58.46	54.87	62.82
F1-Score	28.91	42.10	44.67	18.32	30.12	46.72	63.88	68.80

**Table 8:** Performance metrics for balanced dataset using ratio based data balancing approach with 65:35 ratio.

	Perceptron	MLP Classifier	Naïve Bayes	Logistic Regression	KNN	Decision Tree	Gradient Boosting	XG- Boosting
Accuracy	15.76	74.00	81.28	80.40	76.24	73.92	82.4	86.32
Precision	15.56	31.21	42.10	36.26	27.23	31.75	66.27	56.18
Recall	99.48	55.38	53.33	33.84	31.28	58.46	57.43	59.39
F1-Score	26.92	39.92	47.05	35.01	29.11	41.15	61.53	57.74

**Table 9:** Performance metrics for balanced dataset using ratio based data balancing approach with 50:50 ratio.

	Perceptron	MLP Classifier	Naïve Bayes	Logistic Regression	KNN	Decision Tree	Gradient Boosting	Extreme Gradient Boosting
Accuracy	16.72	53.68	74.40	60.08	57.12	64.08	74.64	72.88
Precision	15.77	21.51	33.06	23.79	20.14	26.39	37.76	32.35
Recall	100	74.35	62.54	70.76	58.97	72.82	64.10	67.69
F1-Score	27.25	33.37	43.25	35.61	30.02	38.74	47.52	43.78

In order to find out the best among the implemented algorithms. Table 10 is considered to evaluate the performance on the basis of several performance metrics.

**Table 10:** Criteria to evaluate the performance of machine learning.

S. No	Measuring parameter	Value
1	Accuracy	High
2	Sensitivity	High
3	Specificity	High
4	Precision	High
5	Recall	High
6	NPV	High
7	F1-Score	High
8	FPR	Low
9	FNR	Low
10	PPV	High
11	TNR	High
12	TPR	High
13	FDR	Low
14	FOR	Low

**Figure 2:**
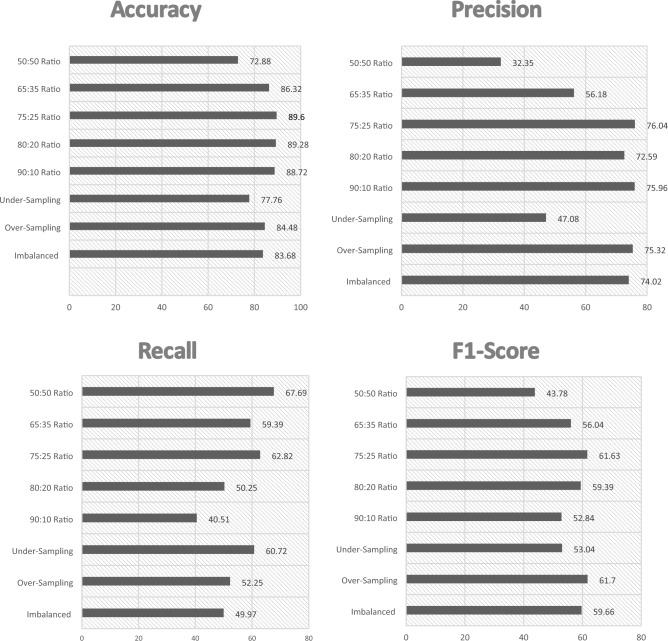
X-G boosting performance metrics for different data balancing techniques.

**Figure 3:**
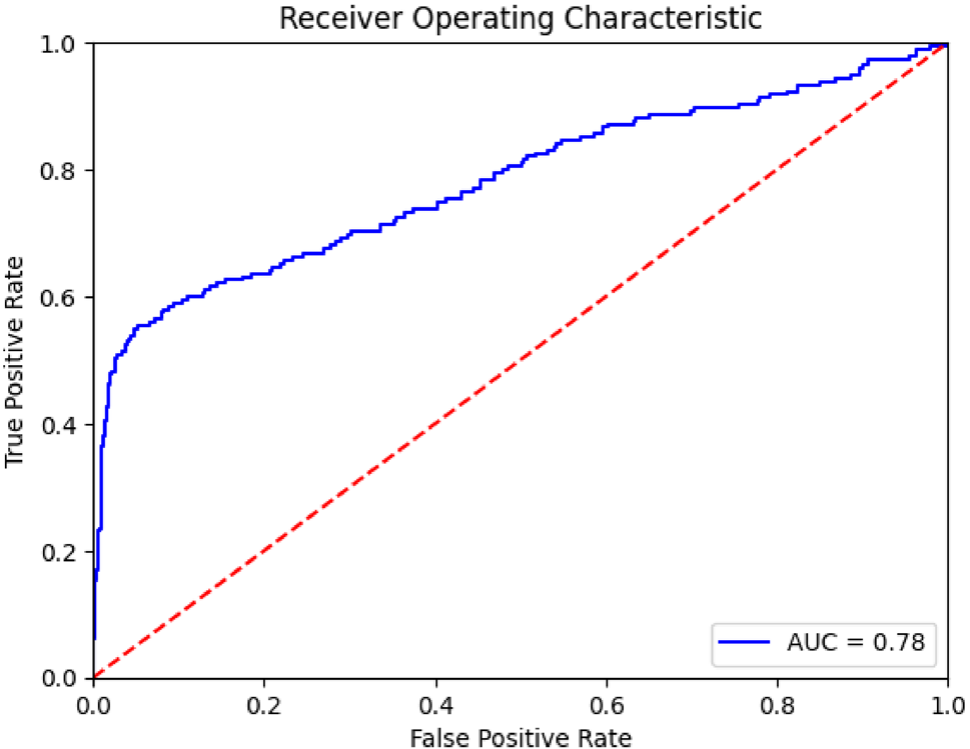
ROC Curve for X-G boosting algorithm at 75:25 ratio.

**Figure 4:**
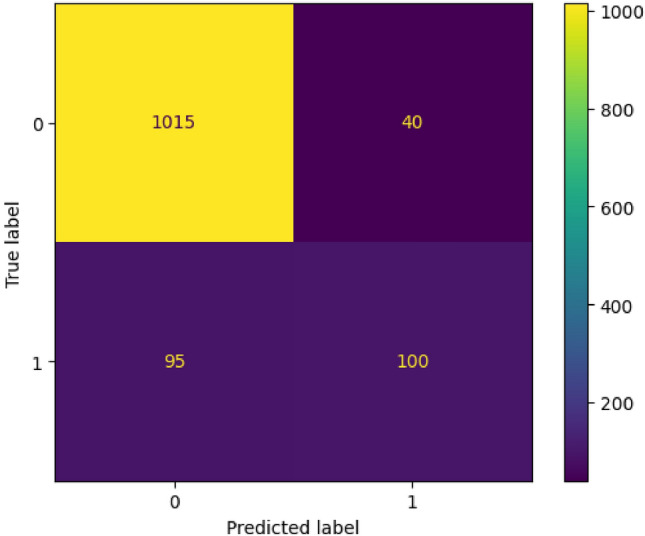
Confusion matrix for X-G boosting algorithm at 75:25 ratio.

**Figure 5:**
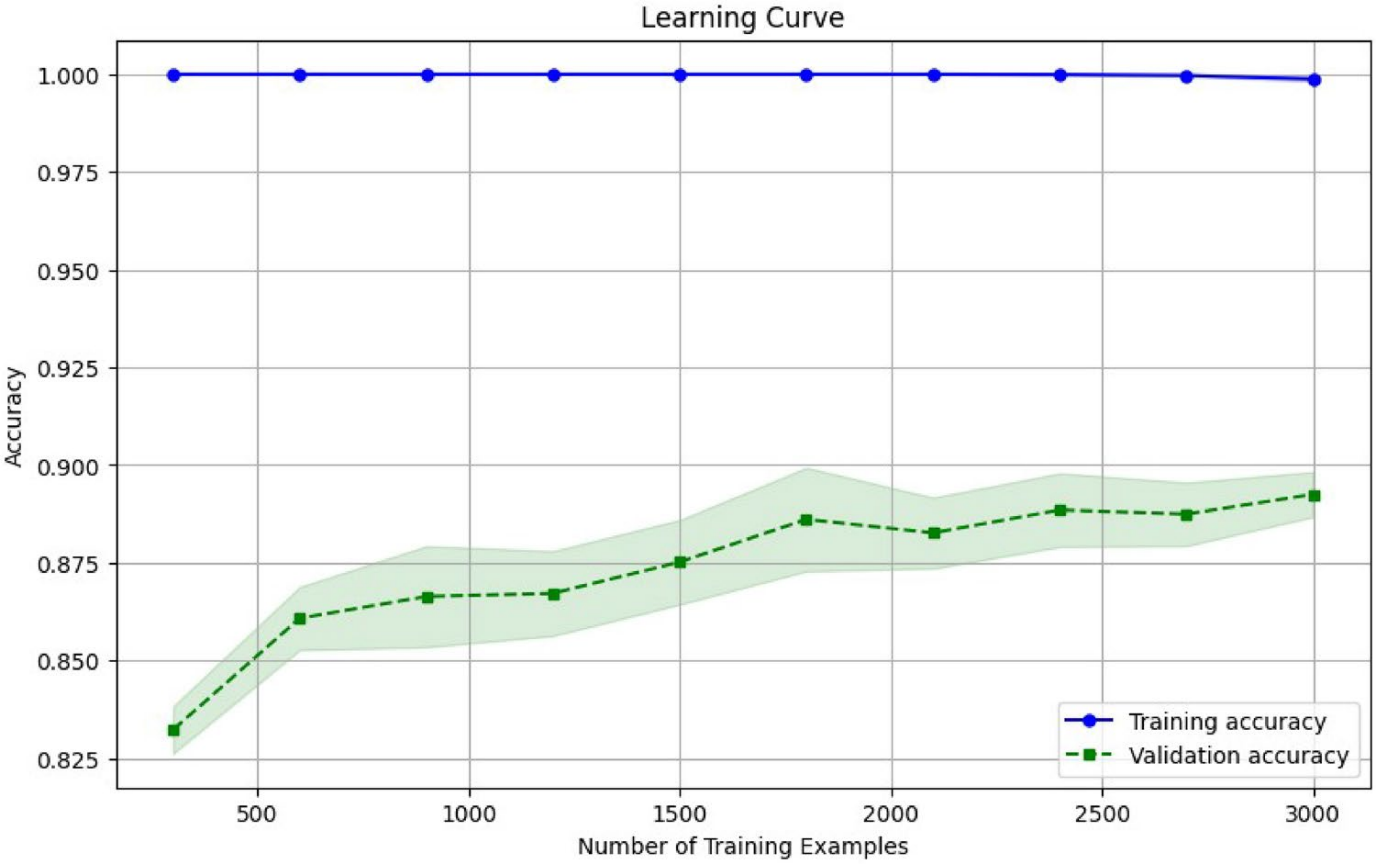
Learning curve of the X-G boost algorithm.

In this work, we have taken four parameters Accuracy, Precision, Recall and F1-Score to find out the best predicting algorithm. As per the criteria given in Table 10. It can be concluded through Rank Aggregation that the best algorithms with maximum highest value results are Gradient Boosting with 10 values and X-G Boosting with 11 highest values, which are both ensemble techniques. Thus, it can be said that the ensemble techniques works better than the standalone algorithms. The different performance metrics for the X-G Boosting are depicted in Fig. 2 below, followed by the ROC curve and Confusion Matrix in Figs. 3 and 4 respectively. The number of training samples and accuracy are taken to depict the learning curve of the X-G Boost algorithm, providing insights into the algorithm’s ability to generalize from the training data. A slope can be seen in the curve in Fig. 5 below that indicates that the algorithm benefits significantly from a greater number of data samples.

The rank aggregation technique is then used to find out the best data balancing technique. The Accuracy and Precision is found to be highest in 75:25 ratio whereas the 50:50 ratio gives the highest Recall and Over-Sampling gives the highest F1- Score. Therefore, it can be concluded that 75:25 ratio-based data balancing technique outperforms all other ratio-based and data resampling based balancing techniques.

Table 11 below gives a comparative analysis of the performance of the approach we found best after data sampling i.e., X-G Boosting with 75:25 ratio and existing algorithms on the same data set.

**Table 11:** Comparison of existing algorithms with X-G Boosting with 75:25 ratio.

Reference	Accuracy	Precision	Recall	F1-Score
^54^	89.10	95.65	16.92	28.76
^59^	83.70	84.20	83.37	83.80
^60^	88.60	80.95	59.44	68.5
X-G Boosting with 75:25 ratio	89.60	76.04	62.82	61.63

## Conclusion and future scope

This research addressed the importance of the customer churn prediction in telecommunication industry. After analysing the area, two gaps were found in the previous literature. (i) Most of the research works done in this field have used the standalone techniques for classifying the customer churn. (ii) The data set available for analysis is imbalanced. Therefore, in order to address the first gap, we have implemented the Ensemble algorithms namely Gradient Boosting and Extreme Gradient Boosting along with the standalone techniques such as Perceptron, Multi-Layer Perceptron, Naive Bayes, Logistic Regression, K-Nearest Neighbor and Decision Tree and compared the performance on the basis of multiple parameters like Accuracy, Precision, Recall and F-Score. Then the rank aggregation approach was used to find out the best classifier among the applied ones. It was found from the experimentation that the Ensemble techniques gives far better results than the standalone ones. To tackle the second research gap, we balanced the dataset using two existing data resampling-based approaches Over-Sampling and Under-Sampling. Furthermore, we have proposed a novel approach called Ratio-based data balancing technique that trains the classification algorithm by taking the target values in a fixed ratio. We evaluated the performance of our proposed approach by taking multiple ratios (i) 90:10 (ii) 80:20 (iii) 75:25 (iv) 65:35 and (v) 50:50. The 75:25 ratio gave the best results with X-G Boost classifier.

Regardless of the novel approach proposed in this research, there are still several concerns in the field that can be addressed in future. This study applied two Boosting based ensemble techniques. In future, other bagging and boosting based ensemble approaches can be applied. Furthermore, the dataset was divided in the ratio of 75:25 for training and testing. Other ratios can also be taken for training the classifiers. The proposed algorithm and existing algorithms are based on sampling of the data however, other cost effective approaches can also be applied to handle the imbalanced data. In this study, we focused on accuracy as the primary criterion for selecting the XGBoost model. However, we recognize the importance of a comprehensive evaluation that includes other critical metrics such as recall and precision. Future work will involve a more detailed analysis using these additional performance metrics to ensure a more robust and well-rounded assessment of model efficacy. This approach will help in identifying the most suitable model for our specific task, ensuring that all aspects of performance are thoroughly evaluated. Last but not the least, the dataset we used to predict the customer churn is not time series based. However, if the analysis is done in real time or on time series based dataset then the classification could be done more accurately and would be more practical.

## Data Availability

The datasets used/analysed during the current study can be made available from the corresponding author on request.
